# Penehyclidine mitigates postoperative nausea and vomiting and intraoperative oculocardiac reflex in patients undergoing strabismus surgery: a prospective, randomized, double-blind comparison

**DOI:** 10.1186/s12871-021-01266-0

**Published:** 2021-02-13

**Authors:** Jiacheng Sun, Xiaofei Cao, Ting Lu, Nan Li, Xinxu Min, Zhengnian Ding

**Affiliations:** Department of Anesthesiology and Perioperative Medicine, First Affiliated Hospital with Nanjing Medical University, Nanjing, 210029 China

**Keywords:** Postoperative nausea and vomiting (PONV), Oculocardiac reflex (OCR), Penehyclidine, Strabismus surgery, Anesthesia

## Abstract

**Background:**

Postoperative nausea and vomiting (PONV) is one of the most frequent complications following strabismus surgery. Penehyclidine, an anticholinergic agent, is widely used as premedication. This study investigated the effect of preoperative penehyclidine on PONV in patients undergoing strabismus surgery.

**Methods:**

In this prospective, randomized, double-blind study, patients scheduled for strabismus surgery under general anesthesia were randomly assigned to either penehyclidine (*n* = 114) or normal saline (*n* = 104) group. Penehyclidine was administrated immediately after anesthesia induction, and normal saline was substituted as control. PONV was investigated from 0 to 48 h after surgery. Intraoperative oculocardiac reflex (OCR) was also recorded.

**Results:**

Compared with normal saline, penehyclidine significantly reduced PONV incidence (30.7% vs. 54.8%, *P <* 0.01) and mitigated PONV severity as indicated by severity scoring (*P <* 0.01). Compared with normal saline, penehyclidine also significantly reduced OCR incidence (57.9% vs. 77.9%, *P <* 0.01) and mitigated OCR severity, as indicated by the requirement for atropine rescue (77.3% vs. 90.1%, *P <* 0.05) and the maximum decrease of heart rate during OCR (23.1 ± 9.4 bpm vs. 27.3 ± 12.4 bpm, *P* < 0.05). The recovery course did not differ between groups.

**Conclusions:**

Penehyclidine administrated after anesthesia induction significantly reduced the incidence of PONV and alleviated intraoperative OCR in patients undergoing strabismus surgery.

**Trial registration:**

ClinicalTrials.gov (NCT04054479). Retrospectively registered August 13, 2019.

## Background

Strabismus surgery is a common ophthalmic surgical procedure, especially in pediatric patients. Postoperative nausea and vomiting (PONV) and intraoperative oculocardiac reflex (OCR) are the most frequent complications in patients undergoing strabismus surgery [1, 2]. The incidence of PONV has been shown between 38 and 68.2% in pediatrics and 45.2% in adults following strabismus surgery [3–6]. Besides unpleasant experience and delayed discharge, PONV can lead to postoperative complications including fluid and electrolyte imbalances, suture tension, esophageal tear, increased intracranial pressure and pulmonary aspiration [1, 7]. OCR, also known as the Aschner reflex, is defined as a decrease in the heart rate (HR) by greater than 20% following eyeball pressure or traction of extraocular muscles [8]. The incidence of OCR during strabismus surgery ranges from 14 to 90% in previous reports [9]. The reflex commonly results in bradycardia and associates with reduced arterial pressure, arrhythmia, asystole, and even cardiac arrest [10]. Both OCR and PONV are the main concerns in patients undergoing strabismus surgery [11].

Penehyclidine is an anticholinergic agent with an elimination half-life over 10 h. As a selective blocker of type 1 and type 3 muscarinic acetylcholine receptor, penehyclidine is widely used as premedication to reduce salivary secretion [12, 13]. Type 3 and type 5 muscarinic acetylcholine receptors have been reported being associated with the development of motion sickness, a risk factor for PONV [14]. Type 1 muscarinic acetylcholine receptor presents at a high level in vestibular system, and anticholinergics block cholinergic transmission from the vestibular nuclei to advanced central nervous system as well as from the medullary reticular formation to the vomiting center [15]. Considering that the muscarinic acetylcholine receptors are involved in the development of PONV through multiple mechanisms, it is possible that penehyclidine may play a role in preventing PONV in patients undergoing strabismus surgery.

This prospective, randomized, double-blind study was designed to identify whether penehyclidine reduces the incidence of nausea and vomiting in patients after strabismus surgery.

## Methods

This study was prospectively approved by the Human Research Ethics Committee of the First Affiliated Hospital with Nanjing Medical University (#2019-SR-238) and a written informed consent was obtained from patient or legal guardian. During the period of 5 months from July to November 2019, a total of 228 consecutive patients aged 3 ~ 65 years, scheduled for strabismus surgery under general anesthesia with American Society of Anesthesiologists physical status I and II were enrolled into the present investigation. All methods were performed in accordance with the relevant guidelines and regulations. This manuscript adheres to the applicable CONSORT 2010 guidelines. The patients with obvious vital organ diseases, motion sickness, previous PONV history, smoking, medication with steroids or proton pump inhibitors, or the patients who did not cooperate with intravenous cannulation were excluded. All cases were prospectively and randomly divided into penehyclidine group and normal saline (NS) group. The primary outcome of our study was the effect of penehyclidine on the incidence of nausea and vomiting during the first 48 h after surgery. The secondary outcome was the possible effect of penehyclidine on the occurrence of intraoperative OCR.

### Study protocol

#### Anesthesia induction

The patients were fasted for over 6 ~ 8 h as a routine, and monitored with ECG, SpO_2_ and non-invasive blood pressure as admitted. After venous access established, lactated Ringer’s solution was infused at a rate of 10 ~ 15 ml·kg^− 1^·hr.^− 1^ throughout anesthesia. Patients were induced with propofol 1.5 ~ 2.5 mg·kg^− 1^ and fentanyl 5.0 μg·kg^− 1^. Cisatracurium 0.15 mg·kg^− 1^ was used to facilitate tracheal intubation. No midazolam or inhalational anesthetic agents were used. Lungs were ventilated with a tidal volume of 7 ~ 10 ml·kg^− 1^ and a frequency of 10 ~ 22 times per minute to maintain end-tidal CO_2_ at the level of 35 ~ 40 mmHg. The fraction of inspired oxygen was maintained at 60%.

#### Anesthesia maintenance

The anesthesia was maintained with infusion of propofol at a rate of 60 ~ 200 μg·kg^− 1^·min^− 1^ and remifentanil at a rate of 0.1 ~ 0.15 μg·kg^− 1^**·**min^− 1^. No muscle relaxant was further added.

#### Anesthesia recovery

Propofol and remifentanil infusion were discontinued as surgery completed. All the patients were spontaneously recovered without the use of neostigmine or atropine to reverse residual muscle relaxant. During the recovery period, the patient who complained of severe pain was treated with parecoxib at a dose of 1.0 mg·kg^− 1^ with an upper limit to 40 mg.

#### Penehyclidine treatment

Simple randomization was achieved by lottery method and the patient was randomly allocated into penehyclidine or NS group with equal chance. Intravenous penehyclidine at the dose of 10 μg·kg^− 1^ with an upper limit to 0.5 mg was administrated immediately after anesthesia induction, and an equal volume of NS served as control. The lots were prepared by a resident, and penehyclidine or NS was given by an attending doctor according to the randomization. Patients were unaware of the treatment groups.

#### Postoperative nausea and vomiting (PONV)

Nausea and vomiting were investigated after tracheal extubation by a resident who was blinded to the treatment. The occurrence of PONV was recorded by direct interview with patient or legal guardian in hospital and by telephone interview after discharge. The severity of PONV was scored using a numeric rank scoring system according to previous method [16]. The scoring system was composed of four levels: 0 = no nausea or vomiting; 1 = nausea but no vomiting; 2 = vomiting once or twice; 3 = vomiting on more than two occasions. The patients with severe PONV were treated with granisetron at a dose of 50 μg·kg^− 1^ with an upper limit to 3.0 mg.

The PONV incidences in different periods, calculated as the percentage of patients who experienced PONV to total number during a specific period, were also analyzed. Some patients may be involved in multiple calculations if they experienced PONV in more than one period.

#### Oculocardiac reflex (OCR)

OCR was defined as a decrease in the heart rate by greater than 20% following eyeball pressure or traction of extraocular muscles [8]. Once OCR was observed, the operation was paused to relieve OCR, and restarted when HR returned to the baseline value. If HR did not recover in 30 s or severe bradycardia (HR < 60 bpm for aged 3 ~ 7 years, and < 50 bpm for more than 7 years old) sustained for over 10 s, intravenous atropine 10 μg·kg^− 1^ with an upper limit to 0.5 mg was administrated.

### Sample size estimation and statistical analysis

Sample size evaluation was performed using PASS software, version11 (NCSS, LLC, Kaysville, UT, USA). On the assumption that the use of penehyclidine will result in a decrease in PONV incidence from 50% in NS group to 30%, 94 cases were needed for each group (α = 0.05 and 1-β = 0.8). With an anticipation of 10% ~ 15% dropout, at least 105 cases were required for each group.

Data analysis was performed using IBM SPSS software, version 24.0 (IBM Corp., Armonk, NY, USA). Continuous variables conforming to normal distribution were expressed as mean ± standard deviation and Student’s two-tailed unpaired *t-*test was used for comparison between two groups. Non-normal continuous variables were expressed as median (interquartile range, IQR) and Mann-Whitney U test was used for comparison between two groups. Categorical variables were expressed as number and percentage, and analyzed by Fisher’s exact test. For ranked data, Mann-Whitney U test was used for comparison. Univariable logistic regression was used to identify the potential risk factors that might affect PONV incidence, and the factors with *P* < 0.10 were included in the multivariable logistic regression. A *P* value *<* 0.05 was considered to be significant.

## Results

### Patients in the investigation

**Fig. 1:**
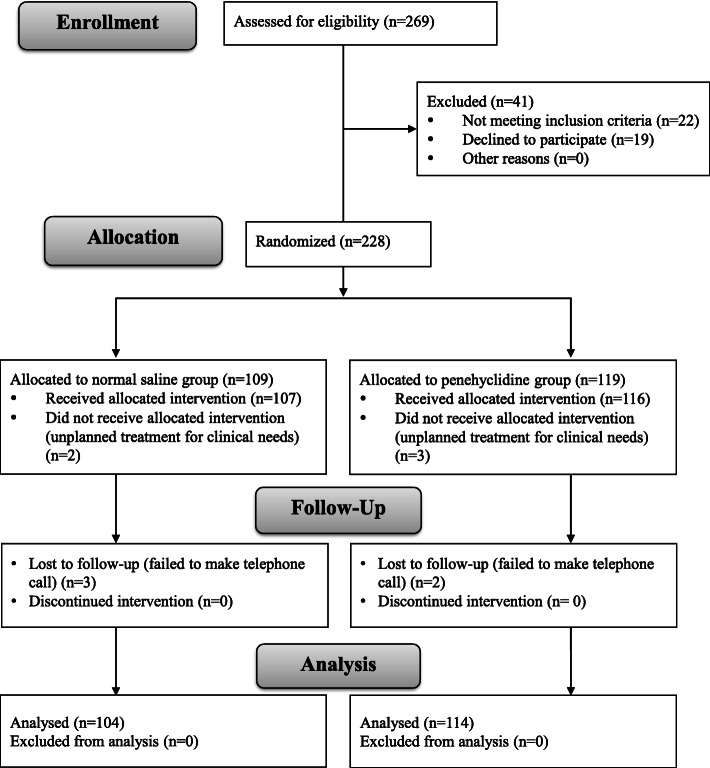
Consort flow diagram. Total 228 patients were randomly allocated to penehyclidine or normal saline group. Among them, 10 patients dropped out due to lost contact or using inhalation anesthesia induction in pediatric patients who did not cooperate with intravenous induction. Finally, 218 patients were analyzed (*n* = 104 in normal saline group and *n* = 114 in penehyclidine group)

During the study period, 228 patients were enrolled into the investigation. Among them, 10 patients dropped out due to lost contact or due to using sevoflurane induction in pediatrics who did not cooperate with intravenous cannulation. Finally, 218 patients were analyzed. The consort flow diagram was shown in Fig. 1.

Between penehyclidine and normal saline groups, patients showed comparable general characteristics, including age, gender, body weight, body height, duration of surgery, duration of anesthesia, unilateral or bilateral operation of eye, and number of operated muscles (Table 1).

**Table 1:** General characteristics of patients

Parameters	Normal saline (*n* = 104)	Penehyclidine (*n* = 114)	*P* value
Age (year)	10 (11)	11 (14)	0.398
Gender (Male/Female)	61/43	57/57	0.222
Body weight (kg)	42.5 (33.4)	47.5 (33.3)	0.412
Body height (cm)	150 (43)	155 (35)	0.656
Duration of surgery (min)	28 (16)	30 (16)	0.668
Duration of anesthesia (min)	64 (17.3)	63.5 (20)	0.631
Unilateral/bilateraloperation	18/86	19/95	1.000
Number of operated muscles	3 (2)	3 (2)	0.753

Age, body weight, body height, duration of surgery, duration of anesthesia and number of operated muscles are expressed as medium (IQR), and compared by Mann-Whitney U test between normal saline group and penehyclidine group. Gender is expressed as numbers of male/female patients and unilateral/bilateral operation is expressed as numbers of the respective patients. Fisher’s exact test was used for comparison between normal saline group and penehyclidine group

### Penehyclidine reduced the incidence and mitigated the severity of PONV in patients undergoing strabismus surgery

**Fig. 2:**
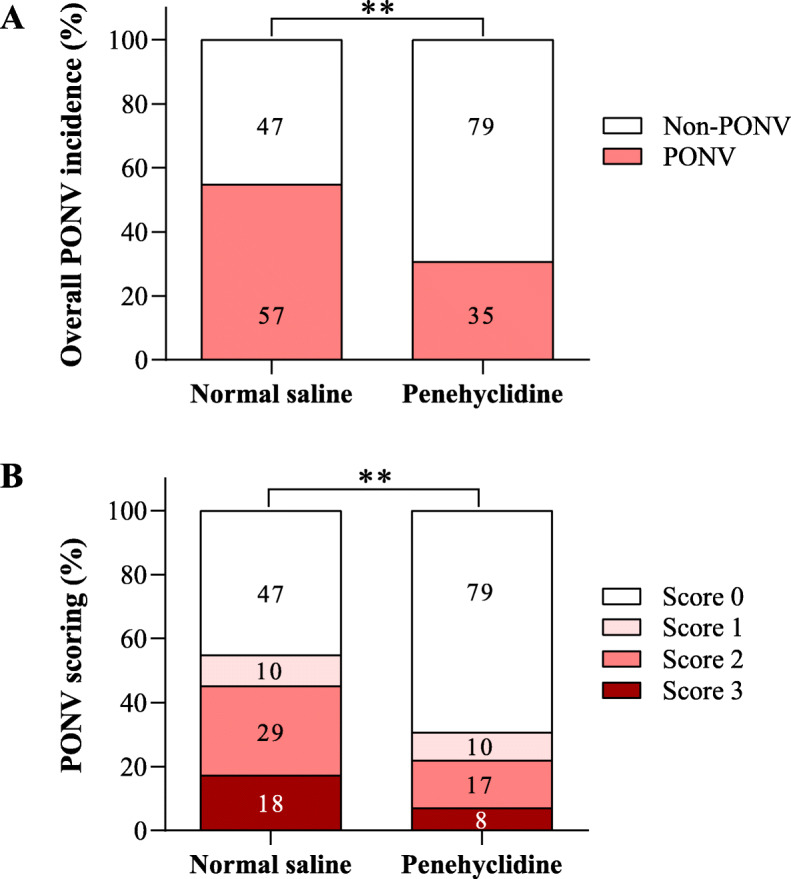
Penehyclidine reduced the incidence and mitigated the severity of PONV in patients undergoing strabismus surgery. **a** The overall incidence of PONV were recorded within 48 h after tracheal extubation. **b** The severity of PONV was scored using a numeric rank scoring system ranging from 0 to 3, wherein 0 represented no nausea and no vomiting and 3 represented vomiting on more than two occasions. ^**^P < 0.01 analyzed by Fisher’s exact test (**a**) or by Mann-Whitney U test (**b**). The numbers of patients are shown in the figures. PONV, postoperative nausea and vomiting

As shown in Fig. 2a, the overall incidence of PONV was 30.7% (35/114) in penehyclidine group and 54.8% (57/104) in NS group (*P <* 0.01). Penehyclidine reduced PONV incidence by 44.0%. Penehyclidine significantly mitigated the severity of PONV, as indicated by the numeric rank scoring system, compared with NS (*P <* 0.01) (Fig. 2b).

### The anti-PONV effect of penehyclidine over time

#### Nausea and vomiting showed high incidence within 6 h after tracheal extubation

**Fig. 3:**
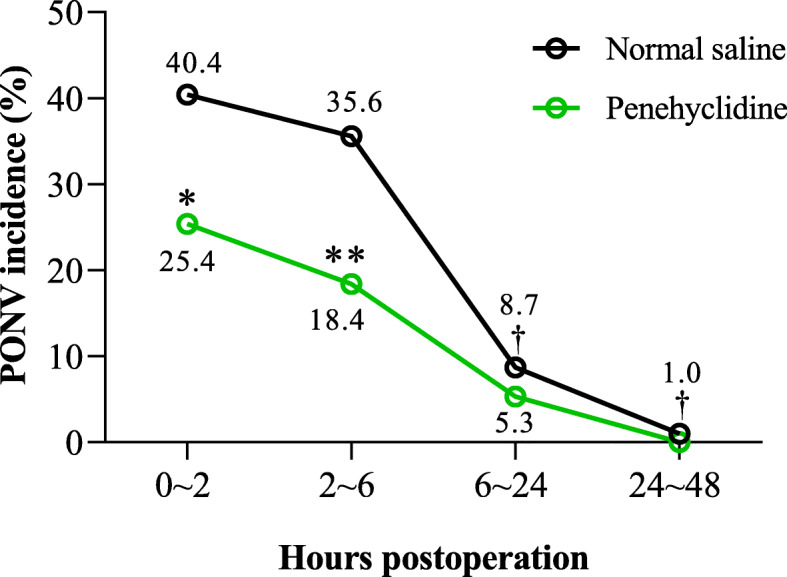
The anti-PONV effect of penehyclidine over time. The PONV incidences in different postoperative periods are shown in the figure. ^†^*P <* 0.01 vs. the incidence during 2 ~ 6 h in normal saline group; ^**^P < 0.01 and ^*^P < 0.05 vs. the time-matched normal saline group, analyzed by Fisher’s exact test. PONV, postoperative nausea and vomiting

To observe the dynamic change of PONV, the PONV incidence during 0 ~ 2, 2 ~ 6, 6 ~ 24 and 24 ~ 48 h after tracheal extubation were analyzed. In NS group, the PONV incidence was 40.4% (42/104) during the first 2 h and remained as high as 35.6% (37/104) during the next period from 2 to 6 h after tracheal extubation. The PONV incidence robustly and significantly decreased during the periods from 6 to 24 h (8.7%, 9/104) and 24 to 48 h (1.0%, 1/104) thereafter, when compared with the incidence during 2 to 6 h after tracheal extubation (*P <* 0.01) (Fig. 3).

#### Penehyclidine significantly reduced the incidence of nausea and vomiting within 6 h after tracheal extubation

In penehyclidine group, the incidence of nausea and vomiting were 25.4, 18.4, 5.3 and 0% in the periods of the first 2 h, 2 ~ 6, 6 ~ 24 and 24 ~ 48 h after tracheal extubation, respectively. PONV incidence in the periods of the first 2 h and 2 ~ 6 h were significantly reduced by 37.0 and 48.2% when compared with the time-matched PONV incidence in NS group (*P <* 0.05 or 0.01) (Fig. 3). No significant difference in PONV incidence was observed between penehyclidine group and NS group in the periods of 6 ~ 24 and 24 ~ 48 h.

### Penehyclidine independently reduced PONV risk

To exclude the confounding factors which potentially interfere the anti-PONV effect of penehyclidine, logistic regression analysis was conducted. Multivariable logistic regression showed that penehyclidine was an independent protective factor (Odds Ratio: 0.330, 95% confidence interval: 0.178 ~ 0.609, *P <* 0.01). Operation with 4 ~ 6 muscles independently increased PONV risk (Odds Ratio: 3.553, 95% confidence interval: 1.909 ~ 6.615, *P <* 0.01). Gender, age, occurrence of OCR, duration of surgery, and duration of anesthesia were not associated with PONV risk in this study (Table 2).

**Table 2:** Univariable and multivariable logistic regression analysis

Variables		Univariable Analysis		Multivariable Analysis	
		Odds Ratio (95% CI)	***P*** value	Odds Ratio (95% CI)	***P*** value
**Gender**	Male	–	–		
	Female	1.146 (0.668 ~ 1.965)	0.621		
**Age (years)**	3 ~ 17	–	–		
	18 ~ 65	0.540 (0.288 ~ 1.011)	0.054	0.750 (0.377 ~ 1.489)	0.410
**EOM operated (n)**	1 ~ 3	–	–		
	4 ~ 6	3.344 (1.906 ~ 5.868)	< 0.01	3.553 (1.909 ~ 6.615)	< 0.01
**Oculocardiac reflex**		2.224 (1.216 ~ 4.068)	< 0.01	1.558 (0.807 ~ 3.006)	0.186
**Penehyclidine**		0.365 (0.210 ~ 0.636)	< 0.01	0.330 (0.178 ~ 0.609)	< 0.01
**Duration of surgery (min)**		1.012 (0.991 ~ 1.035)	0.263		
**Duration of anesthesia (min)**		1.007 (0.991 ~ 1.022)	0.394		

Univariable logistic regression was performed first and the factors with *P* < 0.10 were included in the multivariable logistic regression. As a result, age, extraocular muscles operated, occurrence of oculocardiac reflex and use of penehyclidine were selected for multivariable logistic regression. *CI* Confidence interval, *EOM* Extraocular muscles

### Penehyclidine reduced the incidence and severity of oculocardiac reflex during strabismus surgery

**Fig. 4:**
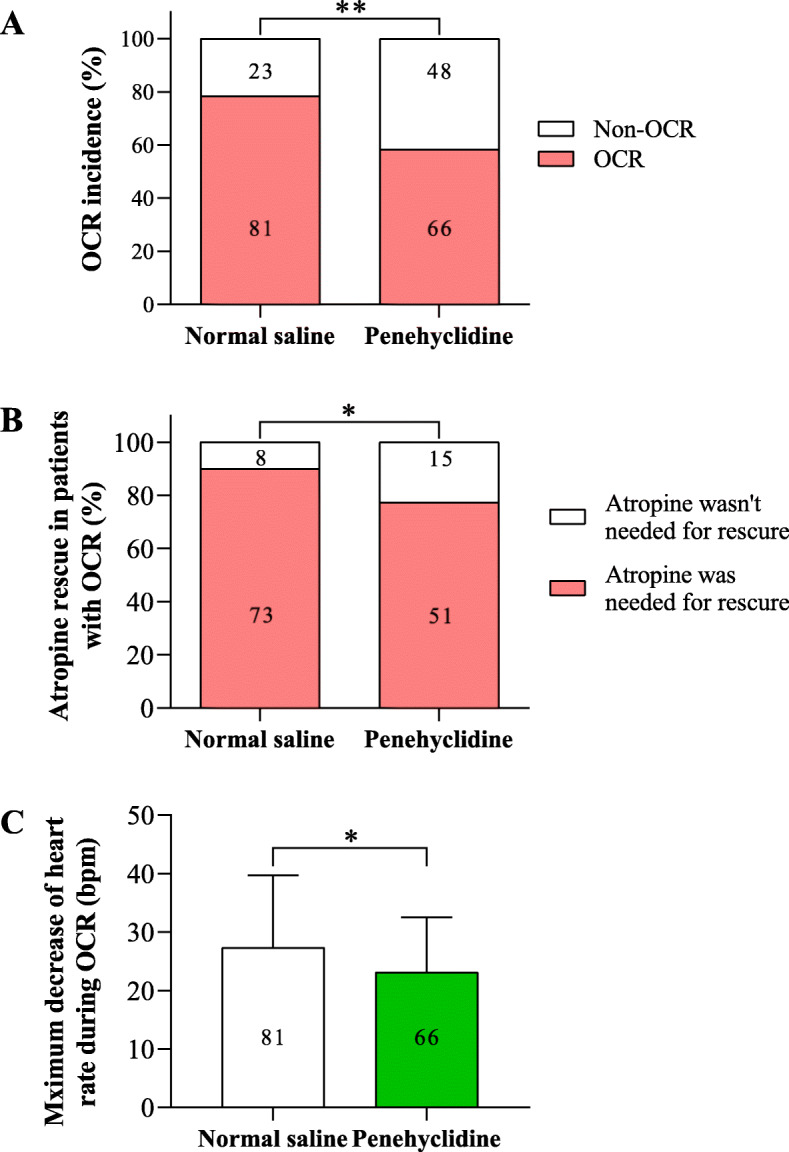
Penehyclidine reduced the incidence and severity of OCR during strabismus surgery. **a** The incidence of OCR in each group. **b** The requirement for atropine rescue in patients with OCR. **c** The maximum decrease of heart rate during OCR. ^**^P < 0.01 and ^*^P < 0.05 analyzed by Fisher’s exact test (**a**, **b**) or by unpaired student *t-*test (**c**). The numbers of patients are shown in the figures. The maximum decreases of heart rate are shown as means ± standard deviation. OCR, oculocardiac reflex; bpm, beats per minute

The incidence of OCR was 77.9% (81/104) in NS group and 57.9% (66/114) in penehyclidine group during strabismus surgery (*P <* 0.01), which was reduced by 25.7% (Fig. 4a). In patients with OCR, atropine rescue was used in 90.1% (73/81) of patients in NS group and 77.3% (51/66) in penehyclidine group (*P <* 0.05), which was reduced by 14.2% (Fig. 4b). Penehyclidine significantly reduced the maximum decrease of heart rate during OCR by 15.4% (23.1 ± 9.4 bpm vs. 27.3 ± 12.4 bpm, *P <* 0.05) (Fig. 4c).

### Penehyclidine did not affect the recovery course after anesthesia

The time from the end of surgery to tracheal extubation, length of stay in the post-anesthesia care unit, use of antiemetics and analgesics, occurrence of severe dry mouth, facial flush, and drowsiness are shown in Table 3**.** No significant difference was detected between groups.

**Table 3:** Postoperative recovery

	Normal saline (*n* = 104)	Penehyclidine (*n* = 114)	*P* value
**Antiemetics used**	4 (3.9%)	1 (0.9%)	0.195
**Analgesics used**	1 (1%)	3 (2.7%)	0.623
**Time to extubation (min)**	25 (16)	25 (13)	0.922
**Length of stay in PACU (min)**	59 (30)	60 (30)	0.732
**Severe dry mouth**	0	0	–
**Facial flushing**	1 (1%)	1 (0.9%)	1.000
**Drowsiness**	0	1 (0.9%)	1.000

Requirement for antiemetics and analgesics, severe dry mouth, facial flushing and drowsiness are expressed as number (%), and compared by Fisher’s exact test between normal saline group and penehyclidine group. Time to extubation and length of stay in PACU are expressed as medium (IQR) and compared by Mann-Whitney U test between groups. PACU, post-anesthesia care unit

## Discussion

The main finding in this study is that penehyclidine administration after anesthesia induction significantly mitigated the incidence and severity of both postoperative nausea and vomiting and intraoperative oculocardiac reflex in patients undergoing strabismus surgery.

PONV is a common complication after general anesthesia and strabismus surgery. The risk factors for the development of PONV relate to patients, anesthetic techniques, and type of surgery [17, 18]. Female, non-smokers, history of PONV or motion sickness, and use of opioids are the most common risk factors [18]. The inhalational anesthetics, ketamine, and etomidate increase the incidence of PONV while the use of propofol, midazolam and free fluid infusion technique are believed to reduce its incidence [1, 19]. For underage patients, duration of surgery over 30 min, age over 3 years and receiving strabismus surgery are independent risk factors for PONV [20, 21]. Many drugs have been used for the management of PONV and the most widely used are 5-hydroxytryptamine (5-HT_3_) receptor antagonists. The Neurokinin-1 receptor antagonists, corticosteroids, butyrophenone and antihistamines are also recommended. However, antiemetic agents raise different concerns just like the risk of QT prolongation in 5-HT_3_ receptor antagonists and the effect on postoperative infection as well as blood glucose levels in corticosteroids [21, 22]. Here in this study, penehyclidine, an anticholinergic agent, significantly reduced the incidence of PONV in patients undergoing strabismus surgery.

The incidence of PONV following strabismus surgery was found to be 54.8% in normal saline group in our result, which is consistent with previous reports. It was also found that PONV mainly occurred in the early recovery period. Penehyclidine administration after anesthesia induction significantly reduced PONV incidence and mitigated its severity.

Unexpectedly, the use of penehyclidine alleviated oculocardiac reflex during strabismus surgery, while penehyclidine was considered having no obvious effect on heart rate [12]. Once stimulated by manipulation, the ophthalmic branch of the trigeminal nerve transports the sensory message to central nervous system, causing impulses exiting the brainstem and transmit to the sinoatrial node. This activates the vagal motor response and ultimately leading to sinus bradycardia, atrioventricular block, ventricular ectopy, ventricular fibrillation or even asystole [10]. Several approaches can be applied to relieve or prevent OCR. Pausing the surgery immediately upon OCR eliminates the pressure on the eyeball or the traction of extraocular muscles, which can alleviate the reflex [10]. Repeated pauses may disturb the process of surgery. Preoperative atropine or glycopyrrolate can attenuate the negative effect of vagus nerve on heart rate during OCR through blocking peripheral type 2 muscarinic receptors in the heart [10, 23]. The main side effect of both agents is undesirable dysrhythmia such as sinus tachycardia. In this study, penehyclidine reduced the incidence of OCR by 25.7%, the requirement for atropine rescue by 14.2%, and the maximum decrease of heart rate by 15.4%. It is unclear whether the effect of penehyclidine on OCR is caused by its intrinsic type 2 muscarinic receptor block effect or its central effects.

The main side effects of penehyclidine include dry mouth and central anticholinergic syndrome, similar to other anticholinergics [15]. Its central sedative effect may delay anesthesia recovery. In our investigation, a dose of 10 μg·kg^− 1^ penehyclidine with an upper limit to 0.5 mg was used. The time to tracheal extubation and the length of stay in the post-anesthesia care unit were found comparable between penehyclidine and NS group. No patient complained of severe dry mouth and no patient developed central anticholinergic syndrome postoperatively. These may be attributed to the limited maximal dose, and that the patients could drink freely after minor surgery [24].

The agents including midazolam, etomidate, neostigmine or inhalational anesthetics were not used in this study in order to avoid potential effects on PONV. The randomization and double-blind technique were strictly followed during the investigation.

## Conclusions

This study identified penehyclidine, a widely used preoperative anticholinergic agent, as an effective protector against postoperative nausea and vomiting and intraoperative oculocardiac reflex in patients undergoing strabismus surgery.

## Data Availability

The data sets generated and analyzed during the current study are not publicly available due to the stipulations of ethics committee to protect individual privacy of patients but are available from the corresponding author on reasonable request.

## Abbreviations

PONV: Postoperative nausea and vomiting
OCR: Oculocardiac reflex
ECG: Electrocardiograph
SpO_2_: Saturation of pulse oxygen
bpm: Beats per minute
IQR: Interquartile range
5-HT_3_: 5-hydroxytryptamine
NS: Normal saline
EOM: Extraocular muscles
